# Predicting outbreaks: a spatial risk assessment of West Nile virus in British Columbia

**DOI:** 10.1186/1476-072X-5-21

**Published:** 2006-05-16

**Authors:** Kaoru Tachiiri, Brian Klinkenberg, Sunny Mak, Jamil Kazmi

**Affiliations:** 1 Department of Geography, University of British Columbia, Vancouver, BC, Canada; 2 Epidemiology Services, British Columbia Centre for Disease Control, Vancouver, BC, Canada; 3 Madrone Environmental Services Ltd., Duncan, BC, Canada

## Abstract

**Background:**

West Nile virus (WNv) has recently emerged as a health threat to the North American population. After the initial disease outbreak in New York City in 1999, WNv has spread widely and quickly across North America to every contiguous American state and Canadian province, with the exceptions of British Columbia (BC), Prince Edward Island and Newfoundland. In this study we develop models of mosquito population dynamics for *Culex tarsalis *and *C. pipiens*, and create a spatial risk assessment of WNv prior to its arrival in BC by creating a raster-based mosquito abundance model using basic geographic and temperature data. Among the parameters included in the model are spatial factors determined from the locations of BC Centre for Disease Control mosquito traps (*e.g*., distance of the trap from the closest wetland or lake), while other parameters were obtained from the literature. Factors not considered in the current assessment but which could influence the results are also discussed.

**Results:**

Since the model performs much better for *C. tarsalis *than for *C. pipiens*, the risk assessment is carried out using the output of *C. tarsalis *model. The result of the spatially-explicit mosquito abundance model indicates that the Okanagan Valley, the Thompson Region, Greater Vancouver, the Fraser Valley and southeastern Vancouver Island have the highest potential abundance of the mosquitoes. After including human population data, Greater Vancouver, due to its high population density, increases in significance relative to the other areas.

**Conclusion:**

Creating a raster-based mosquito abundance map enabled us to quantitatively evaluate WNv risk throughout BC and to identify the areas of greatest potential risk, prior to WNv introduction. In producing the map important gaps in our knowledge related to mosquito ecology in BC were identified, as well, it became evident that increased efforts in bird and mosquito surveillance are required if more accurate models and maps are to be produced. Access to real time climatic data is the key for developing a real time early warning system for forecasting vector borne disease outbreaks, while including social factors is important when producing a detailed assessment in urban areas.

## Background

**Figure 1:**
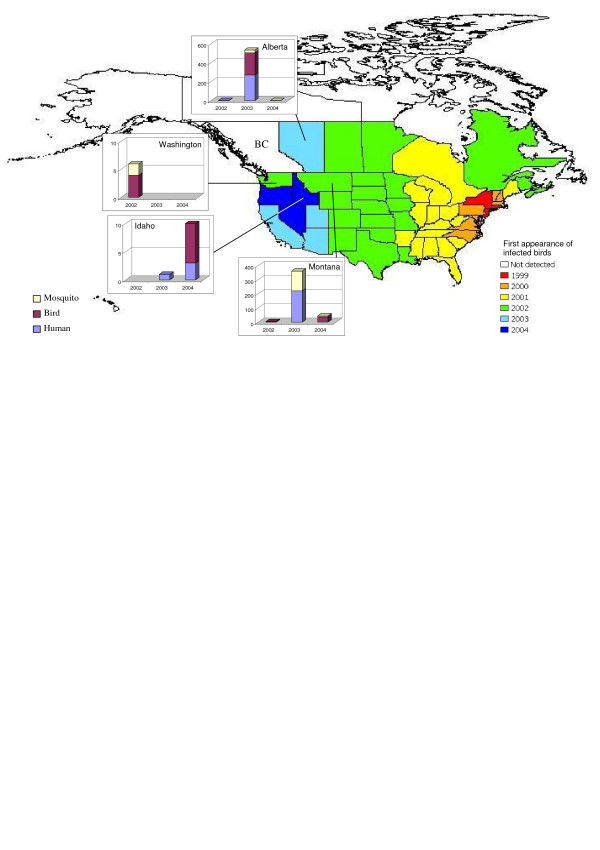
**The spread of the West Nile virus in North America and human cases in BC's surrounding provinces/states**. (Source: [60],[61],[62])

West Nile virus (WNv), a potentially debilitating and fatal mosquito-borne disease first discovered in Uganda in 1937, has recently emerged as a health threat to the North American population. After the initial disease outbreak in New York City in 1999, WNv has spread widely and quickly across North America to every contiguous American state and Canadian province, with the exceptions of British Columbia (BC), Prince Edward Island and Newfoundland (Figure 1). To date, BC has not detected any endemic WNv activity despite operating a comprehensive surveillance program for dead corvids, mosquitoes and human infections. Twenty human cases were identified in BC in 2003, but all were attributed to out-of-province travel [1]. BC is now, however, at the frontier of this disease since WNv has emerged in surrounding regions (Figure 1), including Alberta to the east and Washington, Idaho and Montana to the south. The introduction of WNv into BC appears to be inevitable, since migrating birds from Central America and the southwestern United States, which are now endemic areas for WNv, follow the Pacific Flyway into and through central and coastal BC. Once the virus is introduced, it has the potential to establish itself and spread rapidly, since mosquito species with known competency for hosting and transmitting WNv are present in the province.

The cycle of transmission for WNv consists of the vector (mosquitoes), reservoir (birds) and incidental dead-end hosts (humans and horses) [2]. Infected mosquitoes bite birds and infect them with WNv. Or, alternatively, viremic birds are bitten by naïve mosquitoes and these mosquitoes in turn are infected and pass the virus onto their offspring or other birds that they feed upon. This vector-reservoir cycle can repeat itself several times during the WNv season, resulting in amplified levels of virus in the mosquito and bird populations. Mammals appear to be less important in the transmission cycle [2] as horses, humans and other vertebrates are considered incidental dead-end hosts because viremia in these organisms are not high enough to maintain transmission. Based on the experiences of WNv outbreaks in other North American jurisdictions, *Culex *mosquitoes pose a very high risk for transmitting the virus to birds and humans [3].

In most jurisdictions in North America, dead corvids and mosquito populations are monitored during the WNv season, and act as an early warning system for human infections. In BC, the monitoring program operates between the months of May and October and is coordinated by the BC Centre for Disease Control (BCCDC). Dead corvids and adult mosquitoes from across the province are collected and tested for WNv. The geographic location from where the specimens were submitted is mapped in a Geographic Information System (GIS). Surveillance data in the form of tables, maps and reports are available on the BCCDC website [4].

Spatial risk assessment of WNv, based on mosquito and bird distributions, can help identify areas in the province that are at greatest risk for humans. These high risk areas could, for example, receive higher allocation of government funding to prepare for and respond to the arrival of WNv and associated human illness. This collection of surveillance data, however, only provides point location data that is usually clustered around highly populated human settlements and does not provide risk assessment for areas that lack observations. Fortunately, GIS and geostatistical analysis provide an effective solution to this problem by enabling interpolation of mosquito and bird populations in areas with no observations, based on measured data from existing studies. In particular, spatially and temporally continuous datasets such as vegetation cover, temperature and rainfall, all of which are important elements of mosquito population dynamics, are commonly modelled in this matter [5,6].

In order to assess the seasonal risk of the disease, health agencies in some provinces in Canada are now producing temporal or seasonal maps using temperature data [7,8] to predict mosquito abundance. The current models only consider a simple degree-day model having a single base temperature value (over which a mosquito growth progresses) and a simple degree-day accumulation algorithm (using the condition under which one generation of adult mosquitoes emerge, defined as the product of excess temperature above the base and the number of such days, or heat units) for the entire life of the mosquito. However, it is obvious that such simple model is insufficient to replicate the complex biology of mosquitoes [9], in particular in areas such as BC that have a complex topography. Thus, development of a more sophisticated model, with finer spatial resolution, is necessary if we hope to establish an effective, spatially extensive, early warning system of potential WNv outbreak.

In this article we develop a model of mosquito population dynamics and use this model to produce spatial risk assessment maps of WNv prior to its arrival in BC. This is achieved by combining our mosquito population dynamics model with ancillary data within a GIS. In the maps, forecasted high risk areas for WNv in BC are identified by combining mosquito abundance data with high risk bird abundance data and human population data, which will provide information that can be used to improve the current WNv early warning system. Factors not considered in the current assessment but which can influence the results are also discussed.

Our model is one component of an integrated approach that aims to develop an understanding of the distribution and spread of WNv. Shuai *et al*.'s recent work [10] on the development of a real-time WNv monitoring system for dead corvids in Canada represents another important element of this integrated approach. It is obvious that both technical (*e.g*., web GIS technology) and scientific (virological and ecological) advances are necessary in order to develop a more reliable monitoring system for emerging infectious diseases.

## Results

Using published data on the relation between temperature and mosquito biology (presented in Table 1), we developed a population dynamics model. This model is then incorporated into a spatially-explicit risk assessment model that enables us to map the potential WNv risk.

**Table 1:** Parameters used in the model. (In this table, for degree-days, xx(°C)/YY(dd) where: YY degree-days with the base of xx°C are required to clear the stage, T: daily mean temperature (°C), D: distance from the nearest wetland/lake (m), J: Julian day, h: hours)

		*C. tarsalis*		*C. pipiens*	
Stage and parameter		Parameter value	Source	Parameter value	Source

Start of calculation		3 day mean of T≥ 7.0	Estimated from [45]	3 day mean of T ≥ 7.0	estimated from [45]

	degree-days	9.0°C/20dd	Table 2	7.0°C/30dd	Table 2
Egg	Mortality	0.2%/day(5 ≤ T ≤ 30), (0.02(5-T) + 0.2)% (T<5) (0.02(T-30) + 0.2)% (30<T)/day	[33] (modified)	Same as tarsalis	[33]
	degree-days	7.0°C/170dd	Table 2	9.0°C/150dd	Table 2
Larva	Mortality	(0.5(T-20)^2 ^+ 25)%/2stages of Larva and Pupa	Fig. 4B of [35] (modified)	0.4 (J ≤ 180), 0.6(J>180) for 2stages of Larva and Pupa	[36]
	degree-days	11°C/30dd	Table 2	8.0°C/50dd	Table 2
Pupa	mortality	(see above)	Fig. 4B of [35] (modified)	(see above)	[36]
	mortality	(0.24(T-25)^2 ^+ 5)%/day	[33] (modified)	Same as tarsalis	[33] (modified)
Adult	drying	3 days	[23]	Same as tarsalis	([23])
	host seeking	1 day	[23]	Same as tarsalis	([23])
	ovary development	5.0°C/77dd	Table2([63])	9.5°C/65dd	Table2
Laid eggs/gonotrophic cycle		230	[64] (via [33])	105	[47]
Average gonotrophic cycle		1.10	[46]	5.00	estimated from [47]
Weight by distance (D(m)) from wetland/lake		0.002924-0.000792(D-52)/2138 (if D ≤ 2190), 0.002132-0.001636(D-2190)/484 (if 2190<D ≤ 2674), max(0.000496-0.000230(D-2674)/2865, 0) (if D>2674)	Fig. 10a	0.3054 (if D ≤ 2190), 0.3054-0.1003(D-2190)/1860 (if 2190<D ≤ 4050), max(0.2051-0.1341(D-4050)/1987,0) (if D>4050)	Fig. 10b
Mortality by predators and lack of food (total of immature stages)		89% (if J ≤ 182), 86%(if J ≤ 244), 73%(if J>244)	[42]	98%	Adjusted by fitting
Ratio of oviposition (considering diapause)		1 (if J<150), 0.3 (if 150 ≤ J<180), 0.3-0.01(J-180) (if 180<J ≤ 210), 0 (if 210<J)	[43]	(-412 + 27.2*daylength(h)+2.99*T-8)%	[44]

**Figure 2:**
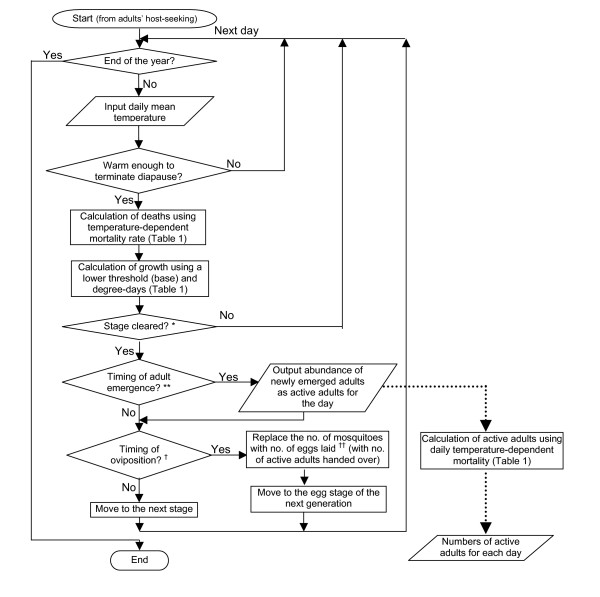
**Flowchart illustrating the mosquito abundance model developed in the study**. * Condition to clear the stages: dd_x _≥ DD_i _(i specifying stages; 1 egg, 2 lava, 3 pupa, 4 adult) where, dd_x _is the accumulated degree-days till day x; DD_i _is the degree-days required to clear the stage i. dd_x _is calculated as follows: where t_x _is the daily mean temperature of the day x; t_0 _is the lower threshold temperature for dd accumulation (DD_i _and t_i _for each stage and species are presented in Table 1). ** Dependent upon accumulation of sufficient DD plus adult drying and host seeking period. † Dependent upon accumulation of sufficient DD for ovary development (presented in Table 1). †† Calculated as: (no. of female mosquitoes) × (no. of avg. gonotrophic cycles) × (avg. no. of eggs per gonotrophic cycle)

**Figure 3:**
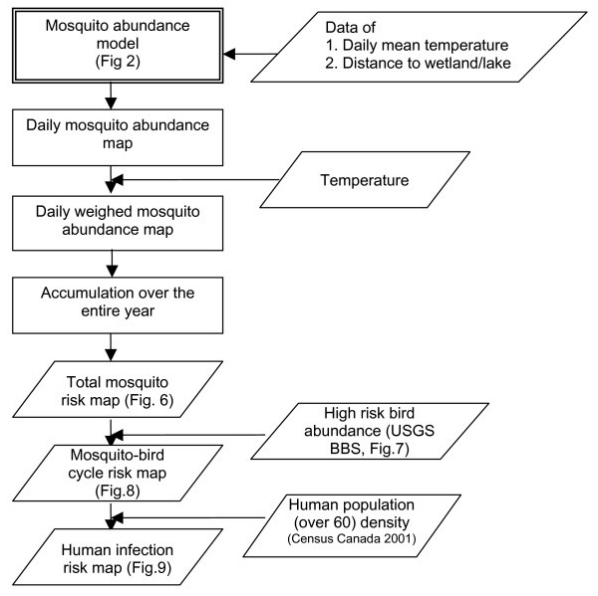
Flowchart illustrating the WNv risk assessment methodology used in the study.

**Figure 4:**
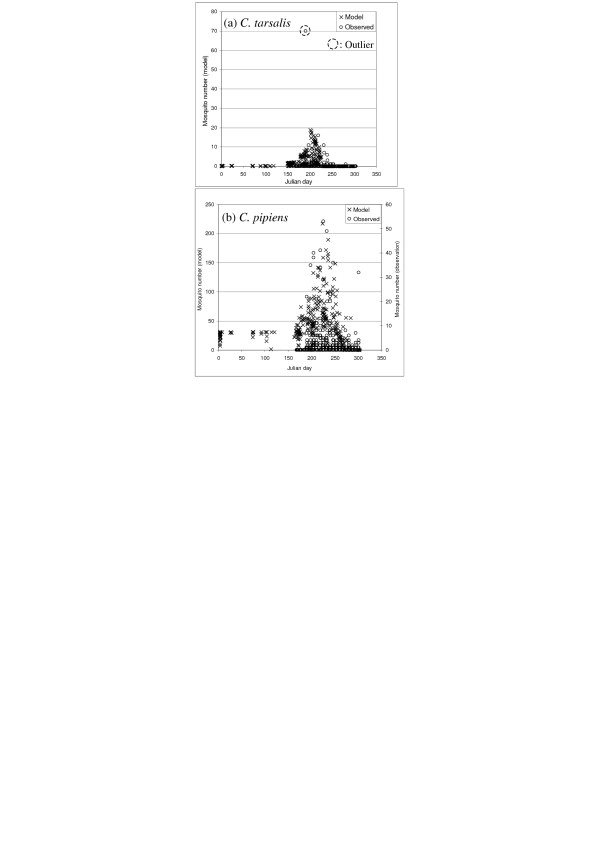
**A comparison of the model outputs and the observed mosquito numbers**. (a) *C. tarsalis *and (b) *C. pipiens*. Data for all traps are mapped together. In the model calculations, the initial egg number is set at 100.

Risk assessment maps of WNv in BC using the mosquito abundance model described below (Figure 2) were produced (Figure 3). In order to examine the validity of the model results, the mosquito population dynamics model output that corresponds to the locations of the BCCDC mosquito traps, with the observation data overlain, is presented in Figure 4. By displaying all of the trap data in a single figure the performance of the model, particularly with respect to the seasonal change in mosquito abundance, can be evaluated. For *C. tarsalis *(Figure 4a), our model produces a good fit to the observation data, with the exception of one data outlier. For *C. pipiens*, on the other hand, our model over-predicts the mosquito numbers in comparison to observed numbers (note that different axes are used for the model and observation numbers in Figure 4b).

**Figure 5:**
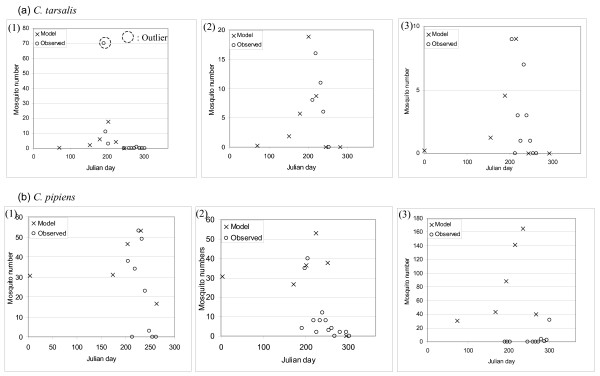
**A trap-by-trap confirmation of the model performance**. (a) *C. tarsalis *and (b) *C. pipiens*. The data for the two stations with largest numbers of mosquitoes observed is presented, along with data from a station in the other main region of concern. For *C. pipiens*, for which b (1) and b (2) traps are enough warm to support mosquitoes in the beginning of January, the initial conditions of the model result in 30 mosquitoes appearing near Julian day 1.

A trap-by-trap check of the model performance (Figure 5) highlights the spatial validity of our model. For each species, data from the two traps that recorded the greatest number of mosquitoes caught in 2003, and another trap from a different region of BC, was used for comparison. The outlier was excluded from our determination of specific model parameters (*i.e*., determining the coefficient value for the distance to wetland/lake). Because the traps with the greatest number of mosquitoes are spatially concentrated (for *C. tarsalis *both are from the Okanagan Valley, and for *C. pipiens *both are from Greater Vancouver), another trap's data is included from the other main region of concern in order to evaluate the model results in a different geographic region (*i.e*., Figure 5a (3), for *C. tarsalis *data from a trap in Greater Vancouver is presented, and 5b (3), for *C. pipiens *data from a trap in the Okanagan Valley is presented). The *C. pipiens *model works well in Greater Vancouver (Figure 5b (1) and (2)) but seriously overestimates population abundances in the Okanagan Valley (Figure 5b (3)), while the *C. tarsalis *model fits both the Greater Vancouver and Okanagan Valley trap abundance data (Figure 5a). These differing model results for the two species highlight the difficulty in predicting the abundance of *C. pipiens*. Some of this difficulty is due to the lack of specific ecological knowledge of *C. pipiens *and the need to use some parameters derived from *C. tarsalis *when modelling *C. pipiens*. However, since *C. pipiens *are typically described as urban mosquitoes commonly found in catch basins and other man-made habitats with stagnant, nutrient rich waters, this decrease in the performance of the model which uses environmental factors is not unexpected. In particular, forecasting *C. pipiens *populations based on ambient air temperature is problematic since the sub-terrain catch basin habitats of *C. pipiens *are considerably cooler than the above-ground temperatures recorded during the WNv season (and therefore overprediction by our model is anticipated).

Since our model significantly overestimates the population abundances for *C. pipiens *in the Okanagan Valley, the following analysis is restricted to considering *C. tarsalis *population dynamics only.

**Figure 6:**
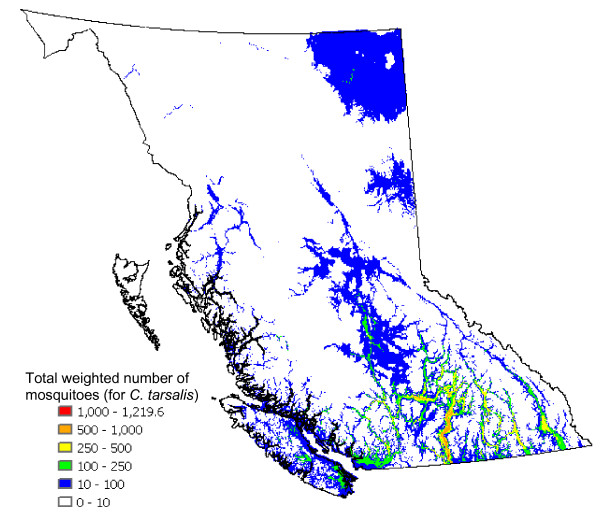
**Annual total of weighed daily mosquito numbers per gird cell (*C. tarsalis *only)**. Weight: 1 for daily mean temperature (T) below 16°C, 2 for 16°C ≤ T<20°C, 3 for 20°C ≤ T<24°C, 4 for 24°C ≤ T<28°C, 5 for T ≥ 28°C (Weight is determined for each day and for each grid cell; see text for a discussion of the methodology used to determine the weights)

The total predicted annual abundance of *C. tarsalis *across the province of BC (Figure 6), derived from the model output, clearly shows the regional nature of their distribution. Since the ability of a vector to transmit WNv is dependent on temperature, as identified by Dohm *et al*. [11], we take that factor into account when creating Figure 6.

**Figure 7:**
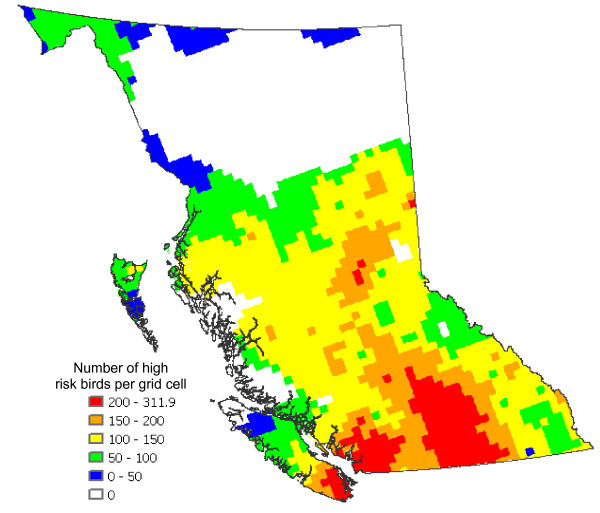
**Total abundance of high risk bird species in breeding season**. The map shows the average number of individual birds considered to be high risk species by the BCCDC (see the additional file for species considered in the map).

**Figure 8:**
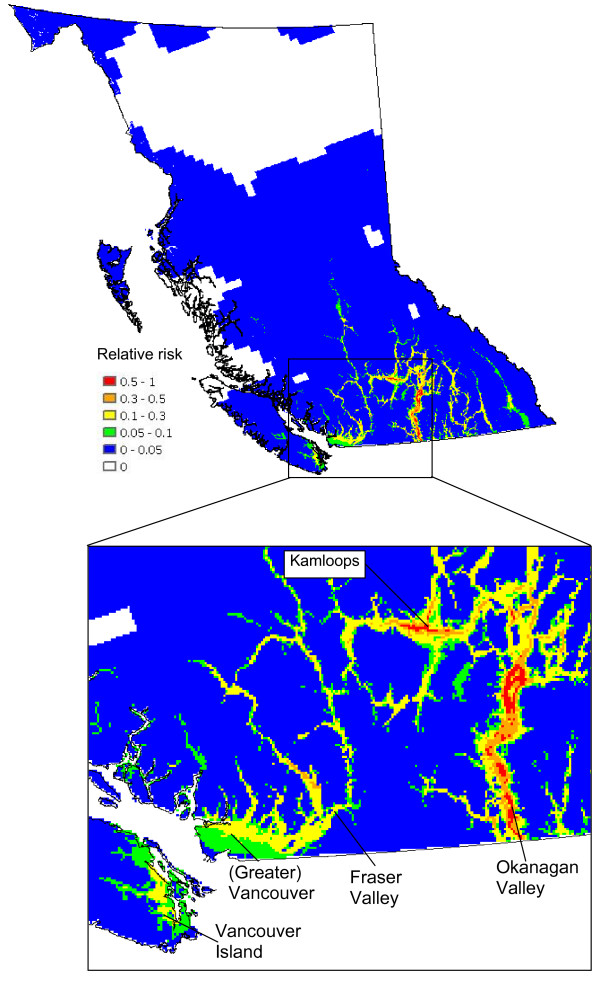
**Total risk of forming a mosquito-bird cycle**. All of BC (upper) and a more detailed look at the high risk areas (lower) (Normalized product of Figures 6 and 7 divided by the maximum value)

The abundance of high risk birds within BC (identified in an unpublished document by the BCCDC) (Figure 7) is estimated by summing the counts of all high risk species (see Additional file 1) from the USGS Breeding Bird Survey (BBS) data for BC. Multiplying the weighted predicted mosquito abundances (Figure 6) by the abundances of high risk birds (Figure 7) we obtain the total WNv risk with respect to mosquito (*C. tarsalis*) and bird populations (Figure 8). This map, normalized by the maximum value, presents the relative risk of a mosquito-bird cycle infected by WNv. The Okanagan Valley and Thompson Region (Kamloops) are identified as the highest WNv risk areas in BC, followed by the Fraser Valley, Greater Vancouver and southeastern Vancouver Island (see the enlarged map in Figure 8).

**Figure 9:**
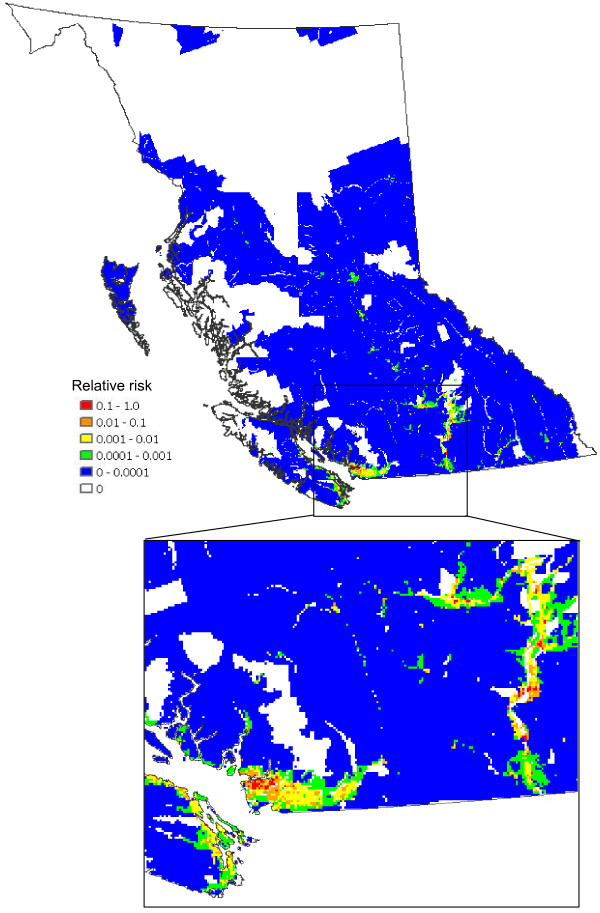
**Risk of human infection**. All of BC (upper) and a more detailed look at the high risk areas (lower) (Normalized product of Figure 8 and human population (≥ 60 years old) density divided by the maximum value)

Finally, multiplying the data presented in Figure 8 with the human population density of people 60 years and older (Census of Canada, 2001) in BC (Figure 9) shows the population-weighted risk of WNv per unit area of land, as contrasted with the data in Figure 8 that evaluates the risk for an individual in each grid cell. Existing studies [12,13] have revealed that older people have a greater risk for infection and showing serious symptoms of the disease. Thus, in creating our final population-at-risk map (Figure 9), an age threshold was applied to the population data prior to producing the population density map. High risk areas in Figure 8 also appear to be of similar high risk in Figure 9, however, Greater Vancouver, due to its high human population density, has an increased relative significance in Figure 9.

## Discussion

The greatest limitation encountered with respect to improving our model's performance is a lack of basic biological information on mosquitoes, particularly for *C. pipiens*, within BC. In this study almost all of the model parameters are obtained from studies on mosquitoes outside of BC (*e.g*., California, Ontario). Detailed information on the biology of BC's mosquitoes, and the specific environmental conditions within which they occur (*e.g*., monitoring temperatures within catch basins), would improve model accuracy.

Additionally, the effect of rainfall, which was not included in our study due to the complex findings of other studies, should be examined and considered in future models if clear relations between rainfall and mosquito population dynamics can be identified. The complex findings of previous studies include the observation that there is a decrease of predators – such as dragonflies – with a subsequent increase in mosquito populations in the year following a significant drought [14], the observation that local concentrations of birds and mosquitoes in limited refuges during droughts can significantly increase the number of birds that are viremic [15], and the complex effects of the drying frequency of wetland ecosystems on mosquito populations, their predators and competitors [16]. Furthermore, since *C. pipiens *is an urban species that often breeds underground in catch basins, better understanding of this species' population dynamics is the key for monitoring WNv illness in urban settings.

Moreover, mosquito ecology and behavioural attributes not considered in our model could be added if more reliable experimental data becomes available. This would include factors such as autogeny (egg development without blood breeding) and mortality during overwintering behaviour. The initial conditions of the current model could be modified and refined with additional knowledge.

Greater numbers of mosquito observations, obtained through more trapping within British Columbia – particularly in the high risk areas identified in this study – could contribute to improved model performance because observational data is important for validating the model's performance and for modifying parameters, as we did in this study. It also should be noted that there are several types of mosquito traps and the differences associated with each trap type may influence the observation results. Vegetation cover close to a trap [17] and the moon phase [18] also influence the trapping results. Therefore, the type, density and detailed location of the mosquito traps should be carefully considered when developing a monitoring program in order to obtain comparable results throughout the province.

A real time monitoring system for WNv is needed in order to quantify the temporal potential risk of WNv in BC. The specific hurdles for developing real time monitoring are related to the availability of climatic and bird data. Daily temperature data is required in developing a mosquito risk map on a real-time basis. At the time of our model development, Environment Canada only provided data for 184 weather stations for 2005, while data from 387 and 363 stations – which can be considered as the potential total active stations – are available for 2003 and 2004. More than half of the stations are not updated on a real time basis, and some immediately updated station data are not quality-controlled (*i.e*., the values are revised later). The root mean-square error (RMSE) of using a limited number of real time stations' data versus using all of the stations' data is evaluated to be 0.80°C for the year, while it is 0.73°C when the temperature is ≥ 5°C, which is the effective temperature for mosquitoes. This error may induce some minor inaccuracy in predicting mosquito abundance. A large uncertainty is associated with the lack of temperature data for the typical habitat of *C. pipiens *(*e.g*., catch basins).

Limitations caused by bird and human population data are also problems one should not ignore. Observational data on migratory birds may enable us to predict the timing of bird migration more accurately, which would be helpful for monitoring the risk of WNv. The nature of Census Canada's population data creates some uncertainty, particularly in sparely populated rural areas covered with large spatial units, however, this is not a serious problem in this study because the high risk areas in BC are consistent with highly populated areas that are covered with fine population survey units.

Social factors, not considered in the current assessment, could be very important for more detailed assessments in urban areas. Ruiz *et al*. [19] report that in addition to the natural factors such as vegetation or geology, the age of housing, the percentage of population that is Caucasian, and the median household income per unit area have a positive relation with WNv cases, while housing density has a negative relation. Ruiz *et al*. [19] also observed that an older person working in the garden during the evening is a good example of a high risk person. An effective public education strategy can decrease their risk of acquiring WNv [20]. Peak mosquito biting activity commences shortly after sunset, and usually peaks during the succeeding 1–3 hours, and lasts until shortly after sunrise [21]. Therefore, people should reduce their activities near wet or heavily vegetated areas around this period, and take personal protective measures such as wearing light coloured long sleeve clothing and applying DEET (mosquito repellent) to avoid mosquito bites. The distribution of road networks can also influence the incidence of WNv. Reisen *et al*. [22] consider this factor as one of the possible infection routes to California in 2003. Importation of mosquitoes on container trucks or private vehicles from Alberta and the United States into BC could potentially occur. Social factors could be easily included in a more detailed GIS-based model.

Finally, the implementation of an integrated pest management plan, which should be targeted to high risk areas, can greatly reduce mosquito numbers and reduce the risk of WNv. While some studies (*e.g*., [23]) attempt to estimate the effect of these activities, developing a sophisticated model enables us to estimate the potential effectiveness of those activities before they are carried out.

The broad areas identified in our study as the highest risk areas are not obviously different from the results produced using simpler models (*e.g*., combination of the presence of the mosquito species capable of WNV transmission, habitat and population). However, population-dynamics based models can provide not only biological rationale to the spatio-temporal change in mosquito abundance, but can also provide more substantive benefits. For example, such a model can present relative risk, with fine spatial resolution, in areas with no mosquito traps or with no dead corvid observations, where simpler models do not work as well. Identifying areas of greatest risk using biologically-relevant methods will assist the concerned agencies in establishing new observation points. Moreover, we can predict changes in WNv risk under different conditions, such as an abnormally hot or cool year, the effect of global warming, the effect of dam construction or reclamation of wetlands, and the differing effects of insecticides (*e.g*., larvacides versus adulticides) on potential outbreaks. While all models start with uncertainty in many parameters, as we collect more data we can validate and reduce the uncertainty associated with the model's parameters. Such improvements will be carried out in the future.

## Conclusion

Spatial risk assessment of the potential for WNv in BC was carried out by developing a sophisticated mosquito abundance model and by combining its output with ancillary data through the use of GIS and geostatistics. First we modeled *C. tarsalis *and *C. pipiens*, but due to the large discrepancy between our model's results and the observed *C. pipiens *numbers, *C. pipiens *was removed from risk assessment. The results show that in terms of mosquito, bird and human populations, the geographic areas at highest risk for WNv in BC are the Okanagan Valley and the Thompson Region (Kamloops), Greater Vancouver, the Fraser Valley, and southeastern Vancouver Island. The resources associated with a WNv early warning system such as mosquito traps and specialist in detecting the virus and in collecting dead corvids, should be concentrated in these areas. Collecting additional information such as that obtained through basic studies on mosquito ecology in BC, as well as continuing efforts in bird and mosquito surveillance, are required for the early detection of WNv and for improving the model, particularly for *C. pipiens*. Access to real time climatic data is also the key for developing a real time early warning system for vector borne diseases, while social factors are important when developing a detailed assessment in urban areas.

## Methods

### Study area and data used

The study area is the province of British Columbia, although northern latitudes and high elevation areas of the province currently do not host mosquito species capable of transmitting WNv [24]. Since the geographic range of mosquitoes may extend northward with warmer climate, should global warming progress, these regions are also mapped in order to provide rough estimates of WNv risk in future years. In BC, there are 46 mosquito species, 17 of which are considered capable of transmitting the virus [25,26]. Among those, *Culex tarsalis *and *C. pipiens *are considered the most important vectors [26]. Accordingly, discussion in this paper is focused and limited to only these two mosquito species.

Data used in this study include climatic data from Environment Canada [27], mosquito observation data from the BCCDC, the GTOPO 30 digital elevation model (DEM) of western North America obtained from the United States Geological Survey [28], breeding bird population data from the USGS [29], and wetland and lake data from the provincial Terrain Resource Information Management Program [30]. Daily maximum and minimum temperature averages from 1971–2000 (only stations that had at least 15 years of records were used in our analyses), and daily temperatures from 2003, are the most important data source used in the analysis. In the model calculations, maximum and minimum temperatures within each day are first interpolated and then the average daily mean temperature is calculated. Daily rainfall data in 2003 are also used for exploring the effect of precipitation. The USGS GTOPO 30 DEM was used to create a 2 × 2 km resolution DEM for the entire province. The North American Breeding Bird Survey (BBS) Summary and Analysis data was used to obtain average bird abundances over the study area. The original data represents the average number of individual birds, over the interval 1994 – 2003, of representative North American species in the breeding season, primarily June, by skilled birders along roadsides (BBS routes). Maps showing averaged abundance by species, created by the USGS using Inverse Distance Weighting from the route data, were used in the study. Wetland and lake data is extracted from the BC Watershed Atlas [31]. Population data, of people aged 60 or greater, was obtained from the Census of Canada 2001. We used the most detailed available data. That is, where Dissemination Area (DA) age-specific data is available (mainly in larger urban centres such as Vancouver, Victoria and Kamloops) it is used. Where such fine-scaled age-specific data is not available, Census Subdivision (CSD) data is used in order to determine the age distribution (the number of people ≥ 60 years old in the DA is calculated as a product of DA's total population and the ratio of people ≥ 60 years old derived from the CSD). In a few cases where neither DA nor CSD data are available, Census Division (CD) data is used in a similar fashion. In total, 7431 DAs, 360 CSDs and 146 CDs were used to identify the ratio of people 60 years of age and older throughout the province. All of the environmental data was gridded to match the DEM (2 × 2 km cell size), and the British Columbia Albers standard projection was used for all maps.

The distance to the sea is calculated by buffering inland from the coast. The 'sea coast' is identified as the area for which the majority of the features within a circle of 20 km diameter is water (the length of the diameter was determined through validation), in order to overcome the impact that the many narrow straits and inlets along the coast of BC have on that distance calculation if just distance from the coast was used. In calculating the distance to the nearest wetland/lake, since it has a shorter range in comparison to distance to the sea, we first use 100 m × 100 m grid cells when calculating the coefficients and then convert them, by averaging, to 2 km × 2 km grids.

### Model development

#### Overview

We developed a population dynamics model of mosquito abundance, linked to environmental factors, whose output is used to produce a risk assessment of WNv. This approach is preferred over a statistical model (*e.g*., a regression model using human cases and possible explaining factors), since, in the case of WNv, the vector-reservoir infection cycle is clear, and to assign too much attention to actual human cases may be misleading since high risk areas may be under-estimated if the human population is low or transient (*e.g*., vacation areas). Furthermore, BC's variable topography and resultant complex temperature variability would ensure that any simple experimental model would not adequately model mosquito distribution and subsequent viral amplification. Finally, since BC has had no human cases that can be attributed to exposure within the province so far, basing a model on human cases is not possible.

The history of mosquito population dynamics models dates back to the 1970s [9,23,32-35]. Most of these models incorporate temperature-dependent growth rates and mortality taken from existing experimental studies as population control factors. Although many studies consider the effect of mosquito density as a controlling factor of mortality, particularly in the egg stage, our proposed model does not include a density-dependent factor as this is difficult to parameterize in the natural environment. Instead, since various conditions can influence the resulting values, and after careful consideration, a number of experimental parameters related to mosquito ecology, obtained from other studies and discussed in the following sections, were utilized in this study. For example, Madder *et al*. [36] mentioned that the difference between their results and Tekle [37] could be due to the difference in the quality and quantity of food, different rearing densities, fluctuations and inaccuracy of temperature controls in the incubators, or an actual difference in genotypes. Also, the vertical transmission (transmission of virus from mother to egg) rate of WNv in *C. tarsalis *and *C. pipiens*, as examined by Dohm *et al*. [38] and Goddard *et al*. [39], ranges between 0 ‰ and 6.9 ‰, and averages around 2–3 ‰. Consequently, we inferred that vertical transmission is not a major route of virus transmission. Instead, we believed that the abundance of adult mosquitoes, especially those which have had multiple gonotrophic (reproductive) cycles, is the primary determinant of the risk of illness through viral amplification in the bird-mosquito cycle.

A flowchart of our model is presented in Figure 2. Contrary to other mosquito population models, which generally provide estimates for one or two isolated populations, our model generates a spatially continuous map-based output for the entire study area. As an initial condition, 100 adult mosquitoes are set for each grid cell (a 2 × 2 km square). The calculation begins with a host-seeking staged adult at the time when the ambient temperature becomes warm enough to terminate mosquito hibernation (diapause). Daily mean temperatures from January 1 are input, and daily growth and death rates of mosquitoes are calculated for each cell.

Developing and verifying the mosquito abundance model parameters, using the trap data, was carried out within Microsoft Excel using Visual Basic for Application (VBA), while the subsequent raster-based GIS calculations for the model were conducted using VBA for ArcGIS. Pre-processing of all the GIS data, including resampling and interpolation, and map-based calculations, including creating risk assessment map using model output, was executed using ArcGIS 9.0. All VBA programs were developed by the authors with the exception of a script, downloaded from the ESRI Support Center [40], to read the value of raster data at the locations of point data.

#### Controlling factors of population dynamics

Mosquitoes have four life stages: egg, larva, pupa and adult. With the exception of extreme conditions, all stages of mosquito maturation occur more rapidly with warmer temperatures. Mosquitoes are unable to regulate their body temperature and, thus, are dependent on the temperature of their surroundings for warmth and growth. The concept of growing degree-days for mosquito population forecasting involves the amount of accumulated heat required for mosquitoes to complete their development from one stage in their life cycle to another. This measure of accumulated heat for development is known as physiological time [41]. Growing degree-days are essentially heat units based on the product of excess temperature (in degrees) beyond the base value and its length (in days). The conditions that are required to complete each life stage of *C. tarsalis *and *C. pipiens*, obtained from existing studies, are summarized in Table 2.

**Table 2:** Base and constant values used for each life stage of *C. tarsalis *and *C. pipiens *derived from existing studies. Base (°C) is the minimum temperature at which mosquitoes in each stage grow, and Constant (dd) is the required amount of degree-days (product of excess temperature above the Base and such days). *: Anautogenous form, **: Autogenous form

*C. tarsalis*						
Stage	Base(°C)	Constant(dd)	R^2^	Remarks	Location	Source
Egg	5.9	37.2	0.98	lab	California	[65]
	9.0	20.9	0.87	fluctuating	California	[65]
						
Larva	7.9	162.6	0.96	lab	California	[65]
	6.2	173.0	0.96	fluctuating	California	[65]
	-40.1	555.6	0.87	fluctuating	California	[65]
	12.7	110.4	0.98	lab	California	[66]
						
Pupa	11.6	29.3	0.99	lab	California	[65]
	189.4	-311.5	0.02	fluctuating	California	[65]
	-76.3	211.9	0.95	fluctuating	California	[65]
	11.7	28.9	1.00	lab	California	[66]
						
Ovarian development	4.9	76.9	0.96	lab	California	[63]
						
*C. pipiens*						
Stage	Base(°C)	Constant(dd)	R^2^	Remarks	Location	Source

Egg	7.4	30.8	0.96	water	Ontario	[36]
	3.7	35.8	0.90	water, fluctuating*	England	[67]
	6.9	29.1	0.92	water, fluctuating**	England	[67]
						
Larva	9.0	107.6	0.85	lab	California	[66]
	4.7	119.2	0.43	lab*	England	[67]
	1.7	171.5	0.38	lab**	England	[67]
	9.5	160.0	0.89		Russia?	[68]
						
Pupa	8.1	34.2	0.85	lab	California	[66]
	8.3	55.9	0.58	lab*	England	[67]
	7.1	57.8	0.61	lab**	England	[67]
						
Ovarian development	9.6	57.8	0.92	field	Ontario	[36]
	10.0	70.9	0.92	lab	Ontario	[36]
	9.1	78.7	0.93	lab	Baltimore	[37]

Studies on mortality show that larva, pupa and adult mortality is similarly temperature-dependent. For example, the estimated mortality curve provided by Eisenberg *et al*. [35], a summary of existing studies, gives a minimum mortality rate of 0.35 at 20°C. Generally, each life stage has an optimal temperature at which mortality is minimized, and mortality increases as the ambient temperature departs from the optimal temperature. One exception is the egg stage, wherein mortality is influenced more by density than by temperature [35]. In nature, however, the predominant factor for mosquito mortality is predation. In a study from California by Reisen *et al*. [42], it was observed that predators are the greatest cause of mosquito mortality: 60–85% of immature *C. tarsalis *mortality was due to predation, while abiotic factors and lack of food resulted in only around 20% mortality. In our model, mortality of *C. tarsalis *due to predation and lack of food is estimated based on the results of Reisen *et al*. [42]. This is 89%, 86% and 73% from January until June, during July/August and during September/October, respectively. For *C. pipiens*, a fixed mortality value of 98%, derived by calculating a best-fitting line to the BCCDC observations, was used since there were no existing studies on mortality caused by predators and lack of food for this species.

Another important factor that influences mosquito abundance is the seasonal change in oviposition (egg laying) behavior that ultimately influences abundance. Mosquitoes have a biological mechanism that increases their procreative power in the early summer. Bennington *et al*. [43] found that the percentage of engorged and gravid *C. tarsalis *females drops rapidly in May, and then stabilizes at around 25% throughout the summer before eventually decreasing to zero at the end of summer (they are fat with stored energy at this point preparing to overwinter). This leads to greater emergence during the summer months.

Futhermore, Madder *et al*. [44] found that temperature, density and day length influence the percentage of adult *C. pipiens *females in diapause (a period of suspended or dormant development, characterized by inactivity and decreased metabolism). Thus, temperature, density and day length also influence mosquito activity.

The date at which the growing degree-days calculations start is also important for the model. Bennington *et al*. [45] reported that the first female *C. tarsalis *in 1954 was observed on Apr. 6 after emerging from hibernation when the soil temperature at a depth of 1–2 m was around 7°C. Due to the absence of soil temperature data, we assumed that a three day average of average daily temperature above 7.0°C is sufficient to break hibernation and initiate the calculation.

An adult female can have multiple gonotrophic cycles. Burdick and Kardos [46] observed the numbers of female *C. tarsalis*, after overwintering, that experienced oviposition. At most four cycles were observed-no mosquitoes survived for a fifth oviposition, and the average number of gonotrophic cycles was 1.10, which is the value used in our model. Tamarina [47] observed an average of 4.08 gonotrophic cycles with 105 eggs per raft for *C. pipiens *(observed until one-half of the sample population died). In our model, which considers the oviposition of survivors, 5.00 is used as a reasonable number of gonotrophic cycles for *C. pipiens*. Also, all eggs (calculated as a product of the average number of gonotrophic cycles and average eggs per raft) from a female adult are laid as soon as the condition for the first oviposition is fulfilled. Daily mortality is not used in the model explicitly since the concept of gonotrophic cycle numbers includes the mortality of adults. However, in determining the total number of active adult mosquitoes, a temperature-dependent daily mortality variable for adults is applied.

All of the parameters used in our model are summarized in Table 1. Note, however, that some of the parameters required to model *C. pipiens *do not exist in the literature, so values derived from *C. tarsalis *studies are substituted. In the following analysis, the daily mean temperature is used in the calculation of degree-days. Also note that in some studies, the temperatures reported were the temperature of the water -in our model we assumed that the average air temperature was an adequate surrogate.

#### Method of interpolating temperature

In this study, the assumption for spatial change in temperature is expressed using the equation that Burrough and McDonnell [48] present for a random variable Z:

Z(x) = m(x) + ε'(x) + ε"     (1)

where m(x) is a deterministic function describing the structural component, ε'(x) is the term denoting locally variable but spatially dependent residuals from m(x), and ε" is residual, spatially independent Gaussian noise having zero mean. That is, m(x) is a stationary (static) part given by some basic topographic factors, and ε'(x) is a dynamic and incidental (unless a complicated numerical large-scale climate simulator is used) part induced by the movement of daily high/low pressures or warm/cool air masses.

To interpolate the daily mean temperature, we modified Odeh *et al*.'s [49] combined regression and kriging method by using Inversed Distance Weighting (IDW) instead of kriging as the geostatistic method. An evaluation of the residuals determined that IDW performed better than kriging for the purpose of this study, and regression-kriging produced better interpolation results than ordinary kriging [49,50], co-kriging [50] and thin plate splines [50].

Jarvis and Stuart [51], in a regression analysis of temperature distribution in England and Wales, used 34 factors and identified elevation, latitude and coastal effect as the most significant contributing factors to maximum and minimum temperature. Among these, elevation was the most important factor, having a negative relation with temperature. As pointed out by Ninyerola *et al*. [52], using the cosine of latitude is more reasonable than latitude itself when modelling temperature. As for the coastal effect, we determined, by plotting the residuals of the regression equation using elevation and cosine of latitude against the distance to the sea, that the following function produced the smallest residuals:

DSEA = d/200 (for d < 200)

    1     (for d ≥ 200)     (2)

Where, DSEA is the coastal effect (0 ≤ DSEA ≤ 1), d = distance from the sea (km).

The regression component (m(x) in eq.1) is calculated based on the long-term (1971–2000) averaged climate data and the geostatistical component (ε'(x) in eq. (1)) is derived from the difference between the data in the year of concern (2003 in this study) and the regression component. Climate data from neighbouring weather stations in Alberta, Yukon Territory and the Northwest Territories were incorporated in our model in order to remove edge effects.

In this study, IDW interpolation is carried out by using ArcGIS (automated by coding of VBA for ArcGIS) with 2 as power and 100 as the number of surrounding stations to be considered in the interpolation.

#### Water dependent factors

**Figure 10:**
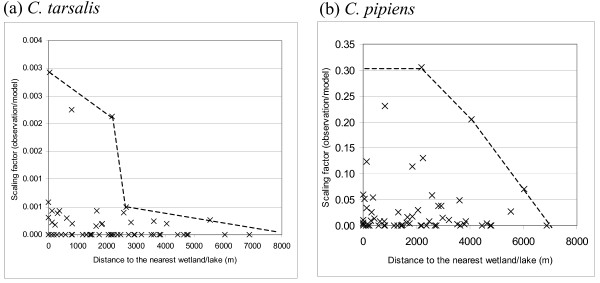
**Relation between scaling factor (observation/model) and distance to the nearest wetland/lake**. (a) *C. tarsalis*, (b) *C. pipiens *Broken lines show relation used in the model. The outlier specified in Figure 4 is not presented.

An overlay of the BCCDC mosquito collection data with the distribution of wetlands and lakes revealed a negative relation between the distance to the nearest wetland or lake and mosquito abundance. This observation is consistent with mosquito biology since mosquitoes require water to lay their eggs, and the first three stages of the mosquito life cycle are aquatic. Additionally, the flight range of mosquitoes is relatively short (unless there is strong wind) so adult mosquitoes tend not to migrate very far from their breeding areas. Bailey *et al*. [53] revealed that a large percentage of *C. tarsalis *in Sacrament Valley of California traveled 2–3 miles (approximately 3–5 km) downwind in one evening and 7 miles (11 km) or more in two evenings, and concluded that it would be realistic to estimate 20–25 miles (32–40 km) as the maximum dispersal distance of individual *C. tarsalis *mosquitoes in this valley, while *C. pipiens *would be limited to a distance of no farther than 1 mile (1.6 km) in an evening [54]. The effect of distance to the nearest wetland or lake in relation to the number of mosquitoes is presented in Figure 10. The negative relationship is clearly evident for *C. tarsalis*, while the *C. pipiens *observations exhibit a peak around 2000 m (which we could not explain). The data outlier in the *C. tarsalis *figure was excluded from our model.

**Figure 11:**
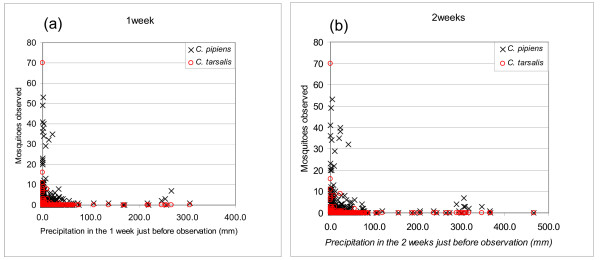
**Relation between rainfall and mosquito abundance**. (a) Against rainfall for 1 week prior to observation and, (b) Against rainfall for 2 weeks prior to observation

A negative relation between trapped mosquito numbers and rainfall recorded at the nearest climatic station is evident in Figure 11. This effect is somewhat counterintuitive to our general understanding of mosquitoes preferring wet conditions, but several studies support this observation. Shaman *et al*. [55] observed that the abundance of *C. pipiens *was negatively correlated with local modeled surface wetness 10 days prior to observation, while other species in their study had a positive relation with the same index. They concluded that this effect was because of the preference by *C. pipiens *for breeding in polluted, eutrophic waters associated with dry conditions. The complex relationship between the abundance of *C. tarsalis *and water-related conditions was examined by Wegbreit and Reisen [56]. Their study revealed that there is no clear relationship between mosquito abundance and rainfall, but there is a strong relation with snow depth, snow water content and river runoff. Based on our observations, where large mosquito sampling numbers are concentrated near zero rainfall events, we conclude that more research is needed prior to establishing such a relation. Therefore, a parameter related to rainfall was not included in our model.

### Bird abundance

The BCCDC (unpublished) has evaluated the risk of bird species in BC that may introduce WNv into the province via migration. Twenty seven (27) species are classified as high risk species (Additional file 1). Recent studies have revealed that global warming may cause early migration for some bird species. Marra *et al*. [57] conclude that for every 1°C increase in spring temperature the timing of migration occurs on average one day earlier. Additionally, Cotton [58] reveals that the timing of migration is dependent on the temperatures at the overwintering ground and breeding site. On the other hand, Both and Visser [59] reported that for species relying on endogenous rhythms, the timing of migration is not affected by climatic change, although the breeding date is affected.

Since we do not have enough information on the temperature-dependency of migration for each species, our model uses the long term average abundance of migratory birds, provided by the USGS, as a factor that increases WNv risk.

### Validation and risk assessment using model output

#### Validation

We first validated our model by comparing its output to the observed mosquito trap abundances (Figure 4). Our model replicated the observed summertime peak abundances, which indicates that our models incorporated mosquito population dynamics successfully. Next, in order to validate the spatially-explicit population dynamics of our models, we performed a trap-by-trap assessment (Figure 5). In Figure 5, for each of *C. tarsalis *and *C. pipiens*, two traps with greatest number of mosquitoes are first selected for validation, and then, in order to examine potential effects of spatial variation, one trap from the other significant region of BC is included (Figure 5a(3) and 5b(3)).

#### Sensitivity analysis

In order to explore the stability of the model output, sensitivity analysis is carried out. All of the results of the analyses, using the data from the two traps with the highest numbers of mosquitoes (*i.e*., the same traps as presented in Figures 5a (1)(2) and 5b (1)(2)), are depicted in Table 3. For *C. tarsalis*, as all of the biological parameters were derived from the published literature, the most uncertain parameter was the soil temperature at which mosquitoes emerge from winter hibernation. Since soil temperature data is not available, air temperature was used as a surrogate. Changing the condition at which diapause is assumed to terminate results in insignificant change in the model's output. For *C. pipiens*, on the other hand, egg and adult mortality as well as the starting condition were all derived from *C. tarsalis *studies and therefore should be checked.

**Table 3:** Sensitivity analysis of the model. Data from the traps presented in Figure 5 are used in these analyses. For the starting condition: a. 7°C for 3 day average, b. 6°C for 3 days, c. 8°C for 3 days and d. 7°C for 2 days. For daily egg mortality (T indicates the mean temperature (°C)): 1. 0.2%/day (5≤T≤30), (0.02(5-T)+0.2)% (T<5), (0.02(T-30)+0.2)% (T >30), 2. 0.2%/day (5≤T≤20), (0.02(5-T)+0.2)% (T<5), (0.02(T-20)+0.2)% (T>20), 3. 0.2%/day (15≤T0≤3), (0.02(15-T)+0.2)% (T<5), (0.02(T-30)+0.2)% (T>30). Totals A, B, C are the annual total mosquito abundance calculated by using an adult daily mortality of: (0.24(T-25)^2^+5)% for TotalA (this is used in this study), (0.24(T-24)^2^+5)% for TotalB, (0.24(T-26)^2^+5)% for TotalC.

*C. tarsalis*							
Trap of Fig. 5(a)1	Start	Max	Date of max	Total			

Final model	a	17.55	Jul.25	326.8			

Model1	b	17.55	Jul.25	326.8			
Model2	c	17.55	Jul.25	326.8			
Model3	d	17.48	Jul.25	325.5			

Trap of Fig. 5(a)2							

Final model	a	18.80	Jul.22	396.3			

Model1	b	18.80	Jul.22	396.3			
Model2	c	18.80	Jul.22	396.3			
Model3	d	18.80	Jul.22	396.3			

*C. pipiens*							

	Start	Egg Mortality	Max	Date of Max	TotalA	TotalB	TotalC

Trap of Fig. 5(b)1							
Final model	a	1	53.00	Aug.18	868.0	1047.0	719.9

Model1	a	2	53.00	Aug.18	868.0	1047.0	719.9
Model2	a	3	52.90	Aug.18	866.4	1045.0	718.6
Model3	b	1	53.00	Aug.18	868.0	1047.0	719.9
Model4	b	2	53.00	Aug.18	868.0	1047.0	719.9
Model5	b	3	52.90	Aug.18	866.4	1045.0	718.6
Model6	c	1	53.00	Aug.18	868.0	1047.0	719.9
Model7	c	2	53.00	Aug.18	868.0	1047.0	719.9
Model8	c	3	52.90	Aug.18	866.4	1045.0	718.6

Trap of Fig. 5(b)2							

Final model	a	1	53.04	Aug.12	1009.4	1186.8	851.1

Model1	a	2	53.03	Aug.12	1009.2	1186.6	850.9
Model2	a	3	52.68	Aug.12	1002.4	1178.7	845.2
Model3	b	1	53.04	Aug.12	1009.4	1186.8	851.1
Model4	b	2	53.03	Aug.12	1009.2	1186.6	850.9
Model5	b	3	52.68	Aug.12	1002.4	1178.7	845.2
Model6	c	1	53.04	Aug.12	1009.4	1186.8	851.1
Model7	c	2	53.03	Aug.12	1009.2	1186.6	850.9
Model8	c	3	52.68	Aug.12	1002.4	1178.7	845.2

Although small changes in the starting condition and egg mortality result in no significant change, it is apparent that altering adult mortality can result in considerable change. Changing the center of the mortality curve by 1C° results in a 15–20 % difference in total annual mosquito abundance. Based on this observation, further investigation in adult mortality of *C. pipiens *is strongly encouraged.

#### Creating risk assessment maps

The methodology presented in Figure 3 is used to create the WNv risk assessment maps presented in Figures 6 to 9. Figure 6 was derived using the following logic. Temperature is known to affect the WNv infection rate [11]. In Figure 1 of Dohm *et al*. [11], the infection rate at four temperatures (18, 20, 26, 30°C) was observed as approximately 30 % for 30 days, 40 %/20 days, 80 %/20 days and 100 %/12 days, respectively. The infection rate X(%) is described as X(%) = e^a(T)t^, where t is time of expose and a(T) is a function of ambient temperature T (°C). If it is assumed, for simplification, that a(T) is a linear function, a(T) is determined as 0.0085T-0.1011 (R^2 ^= 0.88). Substituting 16, 20, 24, 28, 32°C into that equation, the ratios of a(T) at 20, 24, 28, 32°C to that at 16C° are approximately estimated as 2, 3, 4, 5, which are used as the weights in deriving Figure 6 (*e.g*., if the average daily temperature changes from 16°C to 20°C, the rate of increase in the infection rate is doubled).

Figure 7 shows the total abundance of high risk birds.

By multiplying Figure 6 with Figure 7, the risk of forming a mosquito-bird cycle is determined (Figure 8). By dividing the product by the maximum value contained within the map, the relative risk in comparison to the most risky grid cell is evaluated.

The relative risk of human infection is calculated as the product of Figure 8 and the population density of people ≥ 60 years old. This risk is also expressed as a relative one by dividing all the values by the maximum value.

## Competing interests

The authors declare that they have no competing interests.

## Authors' contributions

KT created the model, carried out the analyses and drafted the manuscript. BK conceived, designed and coordinated the study. SM checked the model from the standpoint of mosquito biology and summarized the background of the study. JK participated in conceiving the study and helped to draft the manuscript. All authors read and approved the final manuscript.

## Supplementary Material

Additional File 1 **High risk bird species identified by the BCCDC as potential West Nile virus vectors **(Alphabetically ordered) Click here for file
